# HIV diagnosis period influences ART initiation: findings from a prospective cohort study in China

**DOI:** 10.1186/s12981-021-00379-3

**Published:** 2021-09-09

**Authors:** Tinglong Yang, Xueying Yang, Linghua Li, Huifang Xu, Lirui Fan, Quanmin Li, Xiaoyan Fan, Weiyi Chen, Xuan Du, Chun Hao, Jinghua Li, Yuantao Hao, Jing Gu

**Affiliations:** 1 School of Public Health, Sun Yat-sen University, No. 74 Zhongshan Road 2, Guangzhou, 510080 Guangdong China; 2 South Carolina Smart State Center for Healthcare Quality, Department of Health Promotion, Education, and Behavior, Arnold School of Public Health, University of South Carolina, Columbia, USA; 3 Department of Infectious Diseases, Guangzhou Eighth People’s Hospital, Guangzhou Medical University, Guangzhou, China; 4 Department of HIV Prevention, Guangzhou Center for Disease Control and Prevention, Guangzhou, China; 5 Sun Yat-sen Global Health Institute, Institute of State Governance, Sun Yat-sen University, Guangzhou, China

**Keywords:** HIV, ART, Perceptions, Cohort, China

## Abstract

**Background:**

We estimated the predictive effects of ART-related perceptions on the actual ART uptake behavior among ART naïve PLWH stratified by different time of HIV diagnosis under the new strategy.

**Methods:**

A prospective cohort study was conducted among ART naïve PLWH in Guangzhou, China from June 2016 to June 2017. Cox regression model was used to evaluate the predictive effects of ART-related perceptions on ART initiation among PLWH stratified by different timepoint of HIV diagnosis (i.e., before or after the update of the new treatment policy).

**Results:**

Among 411 participants, 150 and 261 were diagnosed before (pre-scaleup group) and after (post-scaleup group) the implementation of the new strategy, respectively. The ART initiation rate in the post-scaleup group (88.9%) was higher than that in the pre-scaleup group (73.3%) (*p* < 0.001). A significant difference of mean score was detected in each HBM construct between pre- and post-scaleup groups (*p* < 0.05). After adjusting for significant background variables, among all participants, only the self-efficacy [adjusted HR (HRa) = 1.23, 95% CI 1.06 to 1.43, *p* = 0.006], has a predictive effect on ART initiation; in pre-scaleup group**,** all constructs of HBM-related ART perceptions were predictors of ART initiation (HRa = 0.71 to 1.83, *p* < 0.05), while in post-scaleup group, no significant difference was found in each construct (*p* > 0.05).

**Conclusions:**

The ART initiation rate was high particularly among participants who diagnosed after the new treatment strategy. The important role of the time of HIV diagnosis on ART initiation identified in this study suggested that future implementation interventions may consider to modify the ART-related perceptions for HIV patients who diagnosed before the implementation of the new ART strategy, while expand the accessibility of ART service for those who diagnosed after the implementation of the new strategy.

## Introduction

The HIV epidemic remained a global challenge [1]. Based on recent evidence that early antiretroviral therapy (ART) initiation was effective in preventing HIV transmission and slowing the progression of HIV-related diseases as compared to delayed treatment [2–4], WHO proposed the ‘Treatment as Prevention’ strategy in 2013, and recommended immediate ART for all people living with HIV (PLWH) irrespective of their CD4 counts (CD4) in 2015 [5]. Globally the ART coverage was 59% in 2017 [1], and the rate varies across countries (37–72%) [6]. In China, by the end of September 2018, the reported number of PLWH was 850,000, and about 80,000 people are newly diagnosed every year [7]. In June 2016, China launched the new ART strategy which provides free ART irrespective of clinical stage and CD4 count and immediate initiation after diagnosis to all PLWH (hereinafter referred to as ‘the New Strategy’) [8].

Existing literature suggested that the factors associated with ART initiation included socio-demographic characteristics (e.g., age, education level) [9, 10], HIV infection-related characteristics (e.g., route of transmission, CD4 levels) [11, 12], perceptions about ART (e.g., knowledge, treatment intention) [13, 14] and psychosocial factors (e.g., depression, social support) [15–17]. In addition, the evolution of ART guidelines from advocating a cautious application to a near universal approach may also have crucial improvement on ART initiation. A study in Ethiopia found that PLWH who were diagnosed after year 2008 (WHO guideline increased the threshold of CD4 to initiate ART from 200 to 350 cells/μl in 2008) were more likely to initiate ART than those diagnosed before 2008 [18]. Another study in South Africa found that among PLWH with CD4 between 201 to 350 cells/μl, those diagnosed after 2011 (South Africa guideline increased the threshold of CD4 to start ART from 200 to 350 cells/μl in 2011) had higher ART initiation rate than those previously diagnosed [19]. Research in China among HIV positive adolescents [20] and MSM [21] showed similar results. These cohort studies mentioned above confirmed the positive effects of broadened spectrum of eligible individuals on improving timely treatment and ART coverage.

However, there is still a significant number of PLWH who had not initiated ART despite the scale-up strategy, leading to a gap to the UNAIDS 90% ART coverage goal [22]. How these potential ART users (e.g., past and recent diagnoses) perceive the treatment is essential to the implementation of the new strategy, as perceptions are known to be strong predictors of actual behaviors [14]. According to literature review, most existing research among ART naïve PLWH focused on the intention of early ART. For example, research in UK found that 43–47% ART naïve PLWH were willing to start ART [23]; research in Australia found that 72% of HIV positive men who have sex with men (MSM) believed the necessity to start ART as early as possible [24]. Studies in China also reported that early ART was acceptable for over 60% of HIV positive MSM [21, 25, 26]. However, the above studies focused on ART intention and perceptions through cross-sectional designs. So far there is a lack of studies investigating how ART-related perceptions predict actual ART initiation behaviors prospectively after the implementation of new ART strategy.

Behavioral theories help people understand, interpret and improve health-related behaviors [27, 28]. There is a number of behavioral theories being successfully applied in HIV/AIDS area, among which the Health Belief Model (HBM) is one of the most widely used. The HBM has been applied to study late ART initiation [29], ART adherence [30, 31], HIV testing [32], and was also successfully used to guide behavioral interventions [33, 34]. According to HBM, perceived severity, perceived susceptibility, perceived barriers, perceived benefits and self-efficacy are the key constructs, which can be used as potential predictors of ART initiation [27, 28]. How ART naïve PLWH perceive the treatment, whether different subgroups (e.g., PLHW diagnosed before and after the implementation of new strategy) have different perceptions, and how such differences affect ART initiation behavior? Answers to these questions are warranted when implementing the new ART strategy.

This cohort study was conducted right after the New ART strategy (universal ART) being implemented. We aimed to investigate ART-related perceptions, ART initiation behaviors and the predictive effects of perceptions on ART initiation among ART naïve PLWH in China. As the time of HIV diagnosis (before or after the implementation of the new strategy) may affect PLWH’s perceptions toward ART and may lead to different behaviors, we also conducted the subgroup analyses and examined the interactions between perceptions and the time of diagnosis on ART initiation.

## Methods

### Study design and population

This cohort study was conducted from June 2016 to June 2018 in Guangzhou, the capital city of Guangdong Province (China started the new ART strategy since June 1st, 2016) [8]. By the end of 2015, 7311 HIV cases had been reported in Guangzhou and 5056 PLWH (69.2%) were receiving ART (Guangzhou Yearbook, 2016). Six out of 11 districts with the highest HIV prevalence in Guangzhou were selected as the study sites, among which 3 were urban and 3 were suburb districts. All participants were recruited during the first year of the study period and followed up for at least 12 months to assess ART initiation behaviors.

The study population comprised of individuals who were aged ≥ 18 years, confirmed as HIV positive, clinically eligible for ART, never experienced ART before, and could provide informed consent. In total, 439 eligible participants were recruited, of which 411 (93.6%) finished the baseline survey. Ethical approval was obtained from the Ethics Committee of the School of Public Health, Sun Yat-sen University.

### Data collection

According to National Practical Guideline for the Follow-up and Management of PLWH, district-level Centers for Disease Control and Prevention (CDC) or Community Healthcare Centers (CHCs) (sub-district level) are responsible for providing routine care of PLWH in community, including regular follow up and health education [35]. In this study, the trained health care workers at CDC or CHCs contacted all PLWH in the jurisdiction who met the inclusion criteria and invited them to participate in the study. Potential participants were briefed about the study design, ensured the confidentiality issues, and emphasized that refusal to participate would not affect their access to any regular health care services. After informed consent, face-to-face interviews were administered in separate private rooms using structured questionnaires.

## Measurements

### Background information

Background information included socio-demographic characteristics (e.g., age, gender, income) and HIV-related health status (e.g., time of HIV diagnosis, latest CD4 count) (Table 1). Participants were dichotomized by different time of HIV diagnosis into pre-scaleup group (before June 1st, 2016: the implementation of the new strategy) and post-scaleup group (after June 1st, 2016).

**Table 1:** Profiles of participants diagnosed before and after implementing the new ART strategy (n = 411)

	All *n* (%)	Diagnosed before implementing the new strategy *n* (%)	Diagnosed after implementing the new strategy *n* (%)	*P*
Socio-demographic characteristics				
Gender				**0.024**
Male	389 (94.6)	137 (91.3)	252 (96.6)	
Female	22 (5.4)	13 (8.7)	9 (3.4)	
Age (years, mean ± s.d)	32.8 ± 10.6	32.7 ± 9.8	32.9 ± 11.1	0.600
Age (years)				0.603
≤ 25	106 (25.8)	35 (23.3)	71 (27.2)	
25–35	172 (41.8)	67 (44.7)	105 (40.2)	
> 35	133 (32.4)	48 (32.0)	85 (32.6)	
Ethnicity				**0.003**
Han	384 (93.4)	133 (88.7)	251 (96.2)	
Other	27 (6.6)	17 (11.3)	10 (3.8)	
Education				**0.031**
Junior high school and below	113 (27.5)	50 (33.3)	63 (24.1)	
Secondary school	128 (31.1)	50 (33.3)	78 (29.9)	
College or above	170 (41.4)	50 (33.3)	120 (46.0)	
Monthly income (RMB)				0.522
≤ 3000 (434 USD)	154 (37.7)	55 (37.2)	99 (38.1)	
3000–5000 (434–723 USD)	144 (35.3)	57 (38.5)	87 (33.5)	
> 5000 (723 USD)	110 (27.0)	36 (24.3)	74 (28.5)	
Current marital status				0.384
Single	255 (62.0)	99 (66.0)	156 (59.8)	
Married/cohabitation	103 (25.1)	32 (21.3)	71 (27.2)	
Separated/divorced/other	53 (12.9)	19 (12.7)	34 (13.0)	
Household registration				0.304
Guangzhou	94 (22.9)	28 (18.8)	66 (25.3)	
Other cities of Guangdong Province	116 (28.3)	43 (28.9)	73 (28.0)	
Other province	200 (48.8)	78 (52.3)	122 (46.7)	
Residential district				0.384
Urban	288 (70.1)	109 (72.7)	179 (68.6)	
Suburb	123 (29.9)	41 (27.3)	82 (31.4)	
HIV infection-related conditions				
Route of HIV infection				**0.035**
Heterosexual	89 (21.7)	39 (26.2)	50 (19.2)	
Homosexual	301 (73.2)	99 (66.4)	202 (77.4)	
Others (unknown, injecting drug use)	20 (4.9)	11 (7.4)	9 (3.4)	
Latest CD4 cell counts				**< 0.001**
≤ 350	226 (55.0)	54 (36.0)	172 (65.9)	
350–500	121 (29.4)	60 (40.0)	61 (23.4)	
> 500	64 (15.6)	36 (24.0)	28 (10.7)	
Self-reported health status				0.182
Very good/good	168 (40.9)	70 (46.7)	98 (37.5)	
Not good or poor	213 (51.8)	71 (47.3)	142 (54.4)	
Poor/very poor	30 (7.3)	9 (6.0)	21 (8.0)	
HBM-related ART perceptions (mean ± s.d)				
Perceived severity	4.1 ± 0.7	3.9 ± 0.7	4.2 ± 0.7	**< 0.001**
Perceived susceptibility	4.3 ± 0.8	4.1 ± 0.8	4.3 ± 0.8	**0.001**
Perceived benefits	4.2 ± 0.6	4.1 ± 0.7	4.2 ± 0.6	**0.012**
Perceived barriers	3.1 ± 0.7	3.2 ± 0.7	3.1 ± 0.7	**0.028**
Self-efficacy	4.2 ± 0.8	4.0 ± 0.9	4.4 ± 0.6	**< 0.001**

Significant *P* values are in bold

### HBM-related ART perceptions

The HBM-based items of ART perceptions were developed through literature review, in-depth interviews with 15 PLWH, and focused group discussions. The response categories of the questions in the first four constructs were rated on a five-point Likert scale (1 = ‘extremely disagree’, 5 = ‘extremely agree’).

*Perceived severity* was measured by four items, referring to the individual's recognition of the adverse consequences of not being treated in time (e.g., ‘If you do not get treatment in time, the chance of virological failure will increase in the future’). Higher scores represented stronger belief in the serious consequences if not starting ART.

*Perceived susceptibility* in this study was the likelihood of transmitting HIV to one’s HIV-negative sexual partner(s) and was measured by ‘Non-timely participation in treatment will increase the chances of HIV transmission to sexual partners.’ A higher score indicated stronger belief in the chances of HIV transmission if not starting ART.

*Perceived benefits* were measured by six items, referring to the individual’s recognition of the benefits of timely participation in treatment (e.g., ‘Starting ART may increase my CD4 counts effectively’). Higher scores indicated higher perceived benefits of starting ART.

*Perceived barriers* were measured by six items, referring to obstacles that an individual think they may encounter if they initiate ART (e.g., ‘Early ART may lead to early side effects’). Higher scores indicated stronger belief in barriers if starting ART.

*Self-efficacy* was measured by five items, referring to the individual’s confidence in their ability to participate in treatment successfully (e.g., ‘How confident are you that you will schedule your personal and work life in order to start ART?’). Responses were measured on a five-point scale (‘not’, ‘a little’, ‘not sure’, ‘some’ and ‘very’). Higher scores represented a higher level of self-efficacy to start ART immediately.

The above scales showed good internal consistency with Cronbach’s alpha coefficients ranging from 0.79 to 0.92 (Table 2). For item analyses, the responses were dichotomized (e.g., agree/extremely agree, neutral/disagree/extremely disagree).

**Table 2:** HBM-related ART perceptions of participants diagnosed before and after implementing the new ART strategy

	All *n* (%)	Diagnosed before implementing the new strategy *n* (%)	Diagnosed after implementing the new strategy *n* (%)	*P*
Perceived severity^a^ (agree/extremely agree)				
Not starting ART may lead to poor treatment effect	361 (87.8)	117 (78.0)	244 (93.5)	**< 0.001**
Not starting ART may increase the possibility of treatment failure	344 (83.7)	113 (75.3)	231 (88.5)	**0.001**
Not starting ART may accelerate progress of AIDS	370 (90.0)	123 (82.0)	247 (94.6)	**< 0.001**
Not starting ART may lead to drug resistance	287 (69.8)	87 (58.0)	200 (76.6)	**< 0.001**
Perceived susceptibility (agree/extremely agree)				
Not starting ART may increase the possibility of transmitting HIV to my sexual partner	367 (89.3)	129 (86.0)	238 (91.2)	0.102
Perceived benefits^b^ (agree/extremely agree)				
Starting ART may increase my CD4 cell counts effectively	372 (90.5)	133 (88.7)	239 (91.6)	0.333
Starting ART may promote the reconstitution of my immune system effectively	364 (88.6)	130 (86.7)	234 (89.7)	0.359
Starting ART may reduce the possibility of drug resistance	281 (68.4)	95 (63.3)	186 (71.3)	0.096
Starting ART may reduce the possibility of drug complications	378 (92.0)	133 (88.7)	245 (93.9)	0.062
Starting ART may prolong life and reduce the possibility of death	366 (89.1)	131 (87.3)	235 (90.0)	0.398
Starting ART may reduce the possibility of transmitting HIV to others	342 (83.2)	121 (80.7)	221 (84.7)	0.295
Perceived barriers^c^ (agree/extremely agree)				
Early ART may lead to early side effects	162 (39.4)	61 (40.7)	101 (38.7)	0.694
Early ART may lead to early drug resistance	107 (26.0)	38 (25.3)	69 (26.4)	0.806
Early ART leads to early uptake of a lifelong medication burden	235 (57.2)	93 (62.0)	142 (54.4)	0.134
Early ART may interfere with life	142 (34.5)	61 (40.7)	81 (31.0)	0.053
Early ART may cause an economic burden	128 (31.1)	55 (36.7)	73 (28.0)	0.067
Early ART may increase the possibility of disclosure of one’s infection status	194 (47.2)	84 (56.0)	110 (42.1)	**0.007**
Self-efficacy^d^ (some/very)				
How confident are you that you will start ART now?	273 (66.4)	90 (60.0)	183 (70.1)	**0.037**
How confident are you that you will prepare to start ART?	331 (80.5)	102 (71.3)	124 (85.8)	**< 0.001**
How confident are you that you will schedule your personal and work life in order to start ART?	312 (75.9)	100 (66.7)	212 (81.2)	**0.001**
How confident are you to participate in antiviral treatment in the next 6 months?	330 (80.3)	101 (67.3)	229 (87.7)	**< 0.001**
How confident are you to participate in antiviral therapy in the coming year?	341 (83.0)	107 (71.3)	234 (89.7)	**< 0.001**

Significant *P* values are in bold

^a^Cronbach’s alpha coefficient = 0.896

^b^Cronbach’s alpha coefficient = 0.922

^c^Cronbach’s alpha coefficient = 0.792

^d^Cronbach’s alpha coefficient = 0.892

### Primary outcome

The primary outcome was ART initiation, which was defined as the PLWH started ART for the first time. Their ART initiation behaviors were tracked by using the National HIV/AIDS Comprehensive Information System of China’s Disease Prevention and Control information system.

### Statistical analysis

In between-group comparison of the background information (participants diagnosed before or after the new strategy), *t* test, Chi-square or rank sum test was performed accordingly. Kaplan–Meier method was used to calculate the treatment rate of the two subgroups and Log-rank test was used to test the difference.

Univariate cox regression models were fitted to assess the associations between the background variables and ART initiation in all participants. Variables with a *p* < 0.1 were included as candidates in the multivariate stepwise cox regression models, and variables with a *p* < 0.05 were defined as potential confounders. The associations between HBM-related ART perceptions and ART initiation was calculated by both univariate and multivariate analysis (adjusting for potential confounders). The same method was used for subgroup analysis. The results were presented as hazard ratios (HR) with 95% confidence intervals (CI). The significance of interaction effects of time of HIV diagnosis variable on the associations between the HBM-related ART perceptions and ART initiation was tested using multiple cox regression models. A total of 5 interaction models were fitted (each construct of HBM-related ART perceptions × time of HIV diagnosis). SPSS for Windows (version 25.0; SPSS, Chicago, Illinois) was used for statistical analyses. A* p* value < 0.05 was considered statistically significant.

## Results

### Participants profile

Of 411 participants, 150 (36.5%) and 261 (63.5%) were diagnosed before (pre-scaleup group) or after (post-scaleup group) implementing the new strategy, respectively. Most participants were male (94.6%), Han ethnicity (93.4%), single (62.0%), attained college degree or above (41.4%), and self-report as homosexual HIV transmission route (73.2%). The mean age was 32.8 years (standard deviation (SD) = 10.6) and the mean value of their latest CD4 count were 347.8 cells/μl (SD = 182.9). Compared with pre-scaleup group, participants in the post-scaleup group were more likely to be male (96.6% vs. 91.3%, *p* = 0.024), self-report as homosexual HIV transmission route (77.4% vs. 66.4%, *p* = 0.035), attained college degree or above (46.0 vs. 33.3%, *p* = 0.031), and have a lower CD4 count (308.9 vs. 415.5, *p* < 0.001) (Table 1).

### ART-related perception

The difference of mean scores was significant in each construct of HBM-related ART perception between pre- and post-scaleup groups (*p* < 0.05). Specifically, the participants in the post-scaleup group perceived higher severity (*p* < 0.001), higher susceptibility (*p* = 0.001), higher benefits (*p* = 0.012), while lower perceived barriers (*p* = 0.028) and higher self-efficacy (*p* < 0.001) than those in the pre-scaleup group. Regarding specific items of HBM-related ART perception scale, 69.8% to 90.0% of all participants believed that not timely initiate ART will have serious consequences (e.g., treatment failure), especially in the post-scaleup group (76.6% to 94.6%), which were higher than that in the pre-scaleup group (58.0% to 82.0%, *p* < 0.05). Most (89.3%) participants agreed that not timely initiate ART will increase the chance of transmitting HIV to his sexual partner(s). At the same time, most participants (68.4% to 92.0%) perceived benefits of timely ART initiation. There was also a substantial number of participants (26.0% to 57.2%) who had concerns about ART (e.g., worried about the pill burden of taking ART regimen, the exposure of their HIV status, side effects). Compared with the post-scaleup group, the pre-scaleup group were more worried about the exposure of their HIV status when taking medication (56.0% vs. 42.1%, *p* = 0.007). In terms of self-efficacy, 66.4% to 83.0% participants were confident to initiate ART (e.g., scheduled your personal and work life in order to start ART), and the participants in the post-scaleup group (70.1% to 89.7%) had more confidence than those in the pre-scaleup group (60.0% to 71.3%) (*p* < 0.05) (Table 2).

### ART initiation

Among all participants, 342 (83.2%, 95% CI 79.6% to 86.8%) started ART during the follow-up period. The median time interval from baseline enrollment to ART initiation of all the participants was 15 days (ranging from 1 to 758 days), with 65 (95% CI 9.2 to 120.8) days and 12 (95% CI 10.5 to 13.5) days in the pre-scaleup group and post-scaleup group, respectively. The ART initiation rate in the post-scaleup group (88.9%, 95% CI 85.1% to 92.7%) was significantly higher than that in the pre-scaleup group (73.3%, 95% CI 66.2% to 80.5%) (*p* < 0.001).

### The predictive effect of background variables on ART initiation

In univariate analysis of all participants, predictors of ART initiation include: older age, married/cohabited with others, living in the suburb area, higher CD4 levels, self-reported as poor health status, and diagnosed HIV after the new strategy (Table 3). In multivariate analysis (data not tabulated), participants in the post-scaleup group were more likely to initiate ART than those in the pre-scaleup group (multivariate HR (HR_m_) = 2.08, 95% CI 1.65 to 2.64, *p* < 0.001).

**Table 3:** Associations between background variables and ART initiation

	ALL				Diagnosed before implementing the new strategy				Diagnosed after implementing the new strategy			
	(%)	HR_u_	(95% CI)	*P*	(%)	HR_u_	(95% CI)	*P*	(%)	HR_u_	(95% CI)	*P*
Socio-demographic characteristics												
Age (years)												
≤ 25	(79.2)	1.00			(62.9)	1.00			(87.3)	1.00		
25–35	(81.4)	1.08	(0.83, 1.42)	0.564	(68.7)	1.04	(0.62, 1.72)	0.896	(89.5)	1.28	(0.93, 1.77)	0.131
> 35	(88.7)	**1.30**	**(0.98, 1.72)**	**0.068**	**(87.5)**	**1.71**	**(1.02, 2.87)**	**0.043**	(89.4)	1.20	(0.86, 1.69)	0.282
Current marital status												
Single	(80.0)	1.00			(66.7)	1.00			(88.5)	1.00		
Married/cohabitation	(86.4)	**1.30**	**(1.02, 1.67)**	**0.037**	**(87.5)**	**1.99**	**(1.27, 3.11)**	**0.003**	(85.9)	0.99	(0.73, 1.34)	0.963
Separated/divorced/other	(92.5)	**1.35**	**(0.99, 1.84)**	**0.061**	**(84.2)**	**1.66**	**(0.96, 2.87)**	**0.071**	(97.1)	1.16	(0.80, 1.70)	0.436
Residential district												
Urban	(86.5)	**1.00**			(76.1)	1.00			**(92.7)**	**1.00**		
Suburb	(75.6)	**0.75**	**(0.59, 0.96)**	**0.020**	(65.9)	0.72	(0.47, 1.11)	0.138	**(80.5)**	**0.73**	**(0.55, 0.97)**	**0.032**
HIV infection-related conditions												
HIV transmission mode												
Heterosexual	(79.8)	1.00			(82.1)	1.00			(78.0)	1.00		
Homosexual	(85.0)	1.14	(0.87, 1.48)	0.341	**(71.7)**	**0.69**	**(0.46, 1.05)**	**0.087**	**(91.6)**	**1.45**	**(1.03, 2.06)**	**0.034**
Others	(75.0)	0.89	(0.51, 1.55)	0.667	(63.6)	0.53	(0.23, 1.2)	0.126	**(88.9)**	**2.06**	**(0.96, 4.42)**	**0.063**
Latest CD4 cell counts												
≤ 350	(85.8)	1.00			(75.9)	1.00			(89.0)	1.00		
350–500	(81.0)	**0.74**	**(0.58, 0.94)**	**0.014**	(73.3)	0.82	(0.53, 1.25)	0.354	(88.5)	0.89	(0.65, 1.21)	0.448
> 500	(78.1)	**0.63**	**(0.46, 0.86)**	**0.003**	(69.4)	0.69	(0.42, 1.14)	0.151	(89.3)	0.81	(0.53, 1.23)	0.322
Self-reported health status												
Very good/good	(80.4)	1.00			(72.9)	1.00			(85.7)	1.00		
Not good or poor	(84.0)	1.13	(0.91, 1.42)	0.269	(71.8)	1.02	(0.69, 1.50)	0.926	(90.1)	1.10	(0.84, 1.45)	0.498
Poor/very poor	(93.3)	**1.60**	**(1.07, 2.41)**	**0.023**	(88.9)	1.85	(0.88, 3.92)	0.107	(95.2)	1.34	(0.82, 2.18)	0.240
Time since diagnosis of HIV infection		NA										
Within 2 years					(75.8)	1.00						
More than 2 years					(71.4)	0.96	(0.66, 1.4)	0.836				
Time since diagnosis of HIV infection		NA										
Within 15 days									**(91.5)**	**1.00**		
More than 15 days									**(77.1)**	**0.56**	**(0.39, 0.79)**	**0.001**
Time of HIV diagnosis												
Before implementing the new strategy	(73.3)	1.00				NA				NA		
After implementing the new strategy	(88.9)	**2.04**	**(1.62, 2.58)**	**< 0.001**								

Variables which were non-significant in all analyses were not listed in this table, including sex, ethnicity, education, monthly income (RMB), household registration

Significant *P* values are in bold

HR_u_: univariate hazard ratios; CI: confident interval; NA: not applicable

In univariate analysis of pre-scaleup group (Table 3), predictors of ART initiation include: older age, married/cohabitated with others, and self-identified as homosexual transmission. In a multivariate analysis (data not tabulated), participants who were married or cohabitant with others (HR_m_ = 1.96, 95% CI 1.25 to 3.07, *p* = 0.003) were more likely to initiate ART than those who were single. In similar analysis of post-scaleup group (Table 3), participants who were living in suburban (HR_m_ = 0.73, 95% CI 0.55 to 0.97, *p* = 0.032) and those who had more than 15 days of time interval since HIV diagnosis (HR_m_ = 0.56, 95% CI 0.40 to 0.80, *p* = 0.001) were less likely to initiate ART than their counterparts. There was no significant difference for latest CD4 levels in subgroup analyses between pre- and post-scaleup groups.

### The predictive effect of ART-related cognition variables on ART initiation

In univariate analysis of all participants, all constructs of HBM-related ART perceptions were predictors of ART initiation (HR_u_ = 0.86 to 1.38, *p* < 0.05). After adjusting for significant background variables, only the construct of self-efficacy [adjusted HR (HR_a_) = 1.23, 95% CI 1.06 to 1.43, *p* = 0.006] was significantly associated with ART initiation.

In univariate analyses of pre-scaleup group, participants who perceived higher severity (HR_u_ = 1.43, 95% CI 1.10 to 1.86, *p* = 0.007), higher benefits (HR_u_ = 1.66, 95% CI 1.23 to 2.24, *p* = 0.001), and higher self-efficacy(HR_u_ = 1.45, 95% CI 1.15 to 1.84, *p* = 0.002) were more likely to initiate ART, and those who perceived higher barriers (HR_u_ = 0.70, 95% CI 0.53 to 0.91, *p* = 0.007) were less likely to initiate ART. The perceived susceptibility showed no predictive effect of ART initiation. Adjusting for significant background variables, all constructs of HBM-related ART perceptions were still predictors of ART initiation (HR_a_ = 0.71 to 1.83, *p* < 0.05) (Table 4).

**Table 4:** Associations between the HBM-related ART perceptions and ART initiation

	All				Diagnosed before implementing the new strategy				Diagnosed after implementing the new strategy			
	HR_u_ (95% CI)	*P*	HR_a_ (95% CI)	*P*	HR_u_ (95% CI)	*P*	HR_a_ (95% CI)	*P*	HR_u_ (95% CI)	*P*	HR_a_ (95% CI)	*P*
Perceived severity	1.32 (1.13, 1.54)	**0.001**	1.17 (0.99, 1.38)	0.060	1.43 (1.10, 1.86)	**0.007**	1.54 (1.17, 2.02)	**0.002**	1.03 (0.83, 1.27)	0.797	0.99 (0.80, 1.23)	0.957
Perceived susceptibility	1.18 (1.02, 1.37)	**0.029**	1.09 (0.94, 1.27)	0.251	1.24 (0.96, 1.61)	0.096	1.34 (1.02, 1.75)	**0.034**	1.03 (0.86, 1.24)	0.746	1.00 (0.84, 1.19)	0.990
Perceived benefits	1.28 (1.08, 1.52)	**0.005**	1.15 (0.97, 1.38)	0.117	1.66 (1.23, 2.24)	**0.001**	1.83 (1.34, 2.51)	**< 0.001**	0.95 (0.76, 1.19)	0.647	0.92 (0.74, 1.15)	0.459
Perceived barriers	0.86 (0.74, 0.99)	**0.039**	0.91 (0.79, 1.06)	0.216	0.70 (0.53, 0.91)	**0.008**	0.71 (0.55, 0.93)	**0.014**	1.00 (0.84, 1.19)	0.991	0.98 (0.83, 1.17)	0.824
Self-efficacy	1.38 (1.20, 1.60)	**< 0.001**	1.23 (1.06, 1.43)	**0.006**	1.45 (1.15, 1.84)	**0.002**	1.56 (1.23, 1.97)	**< 0.001**	1.11 (0.92, 1.35)	0.272	1.06 (0.87, 1.29)	0.576

Significant *P* values are in bold

HR_u_: univariate hazard ratios; HR_a_: adjusted hazard ratio, adjusting for significant background variables which were significant in the multivariate analyses in Table 3

In univariates and adjusted analyses of post-scaleup group, none of the constructs of HBM-related ART perceptions had predictive effect on ART initiation (*p* > 0.05) (Table 4).

### Interaction

**Fig. 1:**
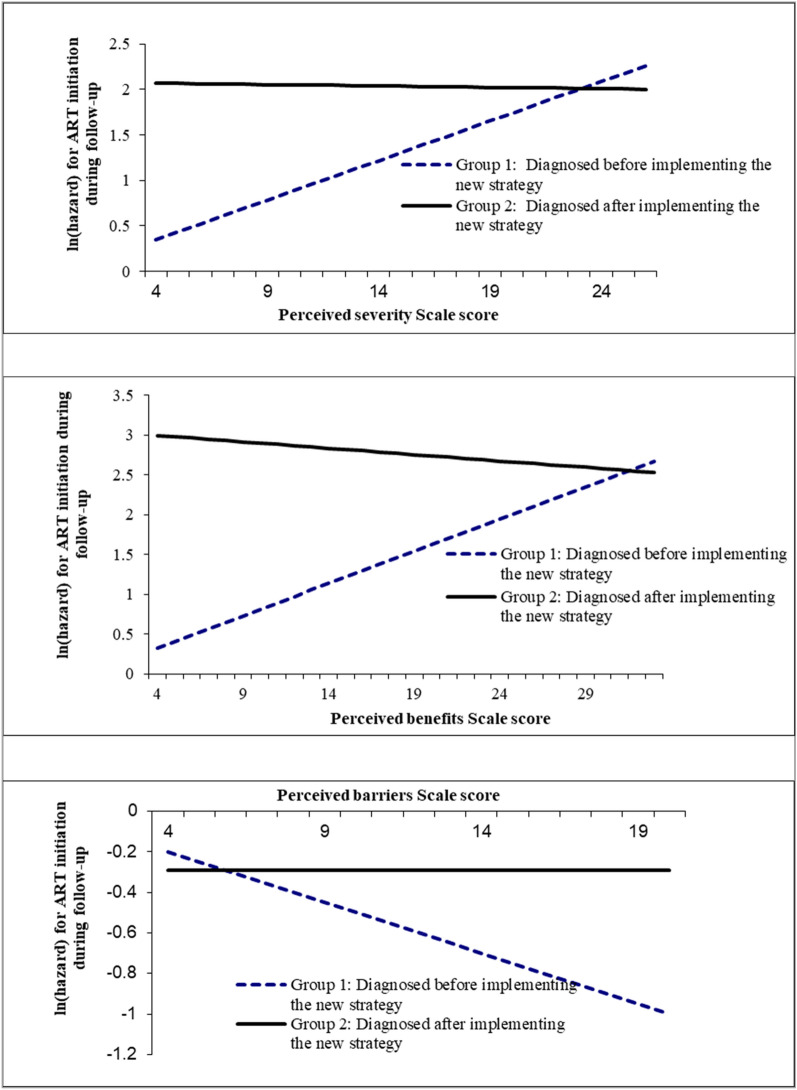
Interaction effects of time of HIV diagnosis (before and after implementing the new strategy) on the associations between the HBM-related ART perceptions (perceived severity scale, perceived benefits scale and perceived barriers scale) and ART initiation

Two out of the five models presented statistically significant interaction effect (Table 5): interaction between time of HIV diagnosis and perceived severity (*β* = − 0.36, *p* = 0.038, Fig. 1), and interaction between time of HIV diagnosis and perceived benefits (*β* = − 0.58, *p* = 0.002, Fig. 1). One out of the 5 models presented marginally statistically significant interaction effect [the time of HIV diagnosis and perceived barriers (*β* = 0.30, *p* = 0.065, Fig. 1)] (Table 5).

**Table 5:** Interaction between HBM-related ART perceptions and time of HIV diagnosis on ART initiation

	Beta	SE (Beta)	*P*
Model 1			
Perceived severity	0.35	0.13	**0.009**
Time of HIV diagnosis^a^	2.08	0.72	**0.004**
Perceived severity * time of HIV diagnosis	− 0.36	0.17	**0.038**
Model 2			
Perceived susceptibility	0.22	0.13	0.096
Time of HIV diagnosis	1.56	0.69	**0.024**
Time of HIV diagnosis * perceived susceptibility	− 0.22	0.16	0.181
Model 3			
Perceived benefits	0.49	0.15	**0.002**
Time of HIV diagnosis	3.06	0.81	**< 0.001**
Time of HIV diagnosis * perceived benefits	− 0.58	0.19	**0.002**
Model 4			
Perceived barriers	− 0.30	0.14	**0.026**
Time of HIV diagnosis	− 0.29	0.53	0.581
Time of HIV diagnosis * perceived barriers	0.30	0.16	0.065
Model 5			
Self-efficacy	0.31	0.12	**0.011**
Time of HIV diagnosis	1.34	0.51	**0.008**
Time of HIV diagnosis * self-efficacy	− 0.24	0.16	0.131

Each interaction model included time of HIV diagnosis, one of HBM-related ART perceptions variables and their interaction term, after adjusting for significant background variables in the multivariate analyses (District, Latest CD4 count)

Significant *P* values are in bold

^a^Time of HIV diagnosis has two levels, before (= 0) and after (= 1) the implementation of new ART strategy

## Discussion

This study was one of the first studies to investigate the ART initiation in a cohort among ART naïve PLWH. The results revealed that ART initiation rate was differentiated by the HIV diagnosis period and a great gap of the median time interval from enrollment to ART initiation was also identified between the two subgroups. In comparison, the ART initiation rate in the post-scaleup group is close to the 90% target, but the pre-scaleup group still has a large gap. Developing targeted interventions for ART naïve PLWH who were diagnosed before the new strategy might be beneficial for achieving the second 90 target-90% ART coverage.

The high levels of HBM-related ART perceptions in this study may possibly be explained by the increased evidence of less side effect and more health benefits of ART [3, 4]. We found that the participants in the post-scaleup group have more favorable ART-related perceptions than those in the pre-scaleup group in all constructs. Several reasons might account for such difference. First, the post-scaleup group received the latest and consistent ART knowledge, while the pre-scaleup group experienced multiple changes in treatment strategies, and ART knowledge obtained may be diverse and inconsistent. Second, the participants in the pre-scaleup group might still under the impression that early treatment was harmful to health and may lead to early drug resistance even if the treatment strategy changed. It may be hard to change the established perception. Third, due to the possible inadequate health promotion of the new strategies, the previously diagnosed PLWH might not comprehensively and accurately understand the new strategies. Therefore, it is necessary to conduct in-depth qualitative and quantitative research to understand the perceptions of HIV patients who diagnosed before the new strategy and to guide the future intervention designs to promote treatment.

Previous studies suggested that ART-related perceptions can be modified to promote the actual ART initiation behavior [13, 14, 29, 36–40]. The different perception levels between the two subgroups might primarily contribute to different ART initiations. Therefore, forming favorable ART-related perceptions for the pre-scaleup group may have a positive effect on ART initiation. Future research may consider using HBM for guiding intervention designs [31], such as delivering the information based on the mobile platform [41], designing short and user-friendly articles and vivid videos [42], developing a website and telephone service platform to provide more convenient consultation and support for PLWH [43], promoting advocacy in specific groups through peer support [44, 45], promoting ART initiation by supporting medical staff with knowledge, training and management skills and integrating such skills into PLWH’s routine care service [46].

In addition, we found that HBM-related ART perceptions had different predictive effects on the ART initiation in different subgroups. In the pre-scaleup group, HBM-related ART perceptions have a predictive effect on the ART initiation, but in the post-scaleup group, there was no significant predictive effect. The reason may be that post-scaleup group has reached a higher level of HBM-related ART perceptions and the variation is not enough to explain the treatment behavior, and the pre-scaleup group experienced multiple changes in treatment strategies and the perception variation was large [47, 48]. Therefore, under the new strategy it may be critical to promoting the proper perceptions for the patients who diagnosed before the new ART strategy. For the post-scaleup group, implementing the immediate ART initiation program [49], increasing the accessibility of treatment could promote the ART coverage in suburban area, given that PLWH who were diagnosed with HIV within15 days (91.5% vs. 77.1%) and those living in urban (97.2% vs. 80.5%) have higher treatment rate than others.

We found an interaction effect of the time of HIV diagnosis and HBM-related ART perceptions on ART initiation. According to the interaction diagram, no interaction effect of HBM-related ART perceptions and time of HIV diagnosis was found in post-scaleup group, but such interaction effect was suggested in the pre-scaleup group, which is consistent with the analyses of the effects of HBM-related ART perceptions on treatment behavior in subgroups.

In this study, we didn’t find the predictive effect of CD4 levels on ART initiation among all participants and subgroup analysis. Although we observed that the higher latest CD4 levels had the lowest treatment rate of all PLWH, such association was not significant after adjusting for the time of HIV diagnosis. This result is inconsistent with previous studies which showed that higher CD4 levels was negatively associated with ART initiation [12]. The unobserved treatment disparity of different CD4 groups in initiating ART is quite promising.

This study has several limitations. First, we only analyzed the predictive effects of background variables and HBM-related ART perceptions on ART initiation and did not involve other factors such as psychosocial variables. Future research may consider these aspects. Second, the study defined the time to ART initiation as the interval between baseline enrollment and actual ART uptake, instead of the time interval from HIV diagnosis to ART initiation. It may not be able to directly examine the impact of new strategy on ART uptake behaviors. Third, we analyzed the ART initiation and its predictors in the two subgroups, but the imbalance in the number of cases in two subgroups may cause insufficient efficacy. Finally, the data collected by the baseline survey was self-reported data and there may be reporting bias.

## Conclusions

In summary, ART initiation, HBM-related ART perceptions and predictive effect of perceptions on ART initiation were different among PLWH diagnosed at different time periods. ART coverage was high under the new strategy, especially among participants who diagnosed after the new treatment strategy. However, a gap still exists referring to the 95% treatment rate target in 2030 [50], especially in the pre-scaleup group. The important role of the time of HIV diagnosis on ART initiation identified in this study suggested that future implementation interventions may consider to modify the ART-related perceptions for HIV patients who diagnosed before the implementation of the new ART strategy, while expand the accessibility of ART service for those who diagnosed after the implementation of the new strategy.

## Data Availability

The datasets used and/or analysed during the current study is available from the corresponding author on reasonable request.

## Abbreviations

ART: Antiretroviral therapy
PLWH: People living with HIV
MSM: Men who have sex with men
UNAIDS: Joint United Nations program on AIDS/HIV
HBM: Health Belief Model
WHO: World Health Organization
CDC: Centers for Disease Control and Prevention
CHCs: Community Healthcare Centers
SD: Standard deviation
HR: Hazard ratio
HR_m_: Multivariate HR
HR_u_: Univariate HR
HR_a_: Adjusted HR
